# Rapid Assessment of Ebola-Related Implications for Reproductive, Maternal, Newborn and Child Health Service Delivery and Utilization in Guinea

**DOI:** 10.1371/currents.outbreaks.0b0ba06009dd091bc39ddb3c6d7b0826

**Published:** 2015-08-04

**Authors:** Janine Barden-O'Fallon, Mamadou Alimou Barry, Paul Brodish, Jack Hazerjian

**Affiliations:** MEASURE Evaluation, Carolina Population Center, University of North Carolina at Chapel Hill, Chapel Hill, North Carolina, USA; MEASURE Evaluation, Carolina Population Center, University of North Carolina at Chapel Hill, Chapel Hill, North Carolina, USA; MEASURE Evaluation, Carolina Population Center, University of North Carolina at Chapel Hill, Chapel Hill, North Carolina, USA; MEASURE Evaluation, Carolina Population Center, University of North Carolina at Chapel Hill, Chapel Hill, North Carolina, USA

**Keywords:** ebola, Guinea

## Abstract

****Introduction: ****Since March 2014, Guinea has been in the midst of the largest, longest, and deadliest outbreak of Ebola Virus Disease ever recorded. Due to sub-optimal health conditions prior to the outbreak, Guinean women and children may have been especially vulnerable to worsening health care conditions. A rapid assessment was conducted to better understand how the delivery and utilization of routine RMNCH services may have been affected by the extraordinary strain placed on the health system and its client population by the Ebola outbreak in Guinea.

****Methods: ****Data were collected January-February 2015 in a convenience sample of public and private facilities in areas of the country that were Ebola active, calm and inactive. Monthly data on a number of RMNCH services were collected by facility record abstraction for the period from October 1, 2013 through December 31, 2014. Structured interviews were also held with facility directors and RMNCH service providers.

****Results: ****Data on RMNCH services from forty five public facilities were obtained. A statistically significant decline of 31% was seen in outpatient visits between October-December 2013 (before the Ebola outbreak) and October-December 2014 (the advanced stage of the Ebola outbreak). Service declines appeared to be greater in hospitals compared to health centers. Child health services were more affected by the Ebola epidemic than other assessed health areas. For example, the number of children under five seen for diarrhea and Acute Respiratory Infection (ARI) showed a large decrease over the one-year period in both hospitals (60% for diarrhea and 58% for ARI) and health centers (25% and 23%, respectively). Results also suggest that the negative effects on service availability (such as reduced hours, closures, and service suspensions) are likely to be regional and/or facility-specific. Providers reported a number of improved infection control behaviors as a result of the Ebola outbreak, including more frequent hand-washing and the use of disinfectants. Nevertheless, 30% of interviewed staff had not received any training on Ebola infection control.

****Discussion: ****Although there may be differences in RMNCH service delivery and availability in selected versus non-selected facilities, a large number of indicators were assessed in order to provide needed information on the effects of the Ebola crisis on routine RMNCH service delivery and uptake in Guinea. This information is an important and timely contribution to ongoing efforts to understand and respond to the adverse effects of the Ebola crisis on essential RMNCH services in Guinea.

## Background

Guinea is in the midst of the largest, longest, and deadliest outbreak of Ebola Virus Disease (“Ebola” hereafter) ever recorded. As one of the three most affected countries in West Africa, which also include Liberia and Sierra Leone, Guinea has recorded 3,729 Ebola cases and 2,482 deaths (as of July 1, 2015) since the first case was diagnosed in March 2014.^1^ This mortality rate (67%) is higher than that of either Sierra Leone (30%) or Liberia (45%). The weekly caseload of the disease in Guinea has shown slight fluctuations throughout the epidemic, peaking at 171 cases during the final week of 2014.^1^ This is in contrast to the higher caseloads experienced by Sierra Leone, which peaked at 570 cases in early December 2014, and Liberia, which peaked at 442 cases in early October 2014.^1^ Ebola in Guinea has disproportionally affected adults (persons aged 15–44 and 45+ are three and five times more likely to be infected, respectively, as those aged <14), and individuals living in certain regions of the country, such as Conakry, Guèckèdou, Macenta, and N’Zèrèkorè.^1^

Notwithstanding the severity of the direct effects of Ebola on the health of the population, experts have also been concerned with the indirect effects of the disease on health care services and mortality from other health conditions. Diseases such as malaria, pneumonia, and typhoid may go untreated due to Ebola-related effects on health systems such as closures of clinics, patients who are afraid to visit facilities for fear of contracting Ebola, or because patients with Ebola-like symptoms are turned away from care.^2^^,^ 3 In some areas, certain services, such as vaccinations, have been suspended due to the lack of personal safety equipment, an inability to conduct real-time tests for Ebola, or insufficient staff to meet the additional health burdens caused by the disease. Projections of measles outbreaks six to 18 months after vaccination disruptions from Ebola are estimated to result in 2,000 to 16,000 additional deaths in the three most affected countries.^4^ The exacerbation of weaknesses in the health system has led to calls for more investment in general system strengthening and the development of “resilient” health systems.^5^^,^ 6 ^,^ 7 ^,^ 8

Information on the “indirect” effects of Ebola at the health-facility level is spotty across the three most affected countries. One study using data on inpatient admissions rates and surgery in neighboring Sierra Leone found a 70% drop in the median number of admissions between May and October 2014 among 40 surveyed facilities.^9^ The authors also found a similar 50% drop in the median number of surgeries during the same time period and estimated that 35,000 sick Sierra Leoneans would be excluded from inpatient care from the onset of the Ebola epidemic through the end of 2014 if the low admissions rates continued.^9^

In Guinea, the number of women giving birth in a facility with a skilled birth attendant in the prefectures of N’Zèrèkorè and Conakry fell by 87% from the period of October through December 2013 to the period July through September 2014.^10^ The rate of new and returning contraceptive users who accessed services at a facility or through community distribution in the same prefectures fell by nearly 70% in the same time periods.^10^ It has also been reported that the hospital in Kissidougou was seeing only 12 to 15 patients a day at the end of September 2014, compared to a typical intake of 200 to 250 patients per day.^3^

Guinean women and children may be especially vulnerable to worsening health care conditions. Prior to the Ebola epidemic, a key indicator of maternal health, the maternal mortality ratio, was 650 per 100,000 live births (2012).^11^ Facility-based deliveries were occurring for only 41% of births, the contraceptive prevalence rate was 6%, and child health indicators ranked low: the under-five mortality rate was 101 per 1,000 live births, with malaria as the top cause of death, and full immunizations were received by only 36.5% of children.^11^^,^ 12 There is indication that women were avoiding services due to stigma and fear. Statistics from Matam maternity hospital in Conakry showed a drop in attendance during the advanced stage of the epidemic, with 123 patients for the July through September 2014 quarter, compared to 760 patients for the same quarter in 2013.^2^

Based on such reports, it has been assumed that levels of service delivery for routine reproductive, maternal, newborn, and child health (RMNCH) care have fallen precipitously in some areas over the course of the Ebola outbreak in Guinea. However, no data are currently available to assess the extent to which this may be true across geographic zones and different prefectures, or to inform planning and resource allocation in response to the challenge created by the sudden and severe Ebola outbreak.

## Objectives

A rapid assessment was conducted to better understand how the delivery and utilization of routine RMNCH services may have been affected by the extraordinary strain placed on the health system and its client population by the Ebola outbreak in Guinea. At the time the assessment was undertaken, no such information was systematically available to guide the response of local officials and donors concerned about RMNCH services. This rapid assessment was commissioned by USAID/Guinea as a way to provide a quick yet systematic look at the status of RMNCH service delivery and utilization in selected facilities during the period immediately before the Ebola outbreak, compared to the conditions in Guinea approximately one year later. Specific objectives of the assessment were: (1) to obtain information to better understand the implications of the Ebola crisis on provision and uptake of RMNCH health care services in a sample of health facilities, and (2) to obtain preliminary information from health care providers on implications of the Ebola crisis on routine service delivery and practice.

## Methods

**Study design**

A retrospective facility records review combined with structured interviews with health personnel was carried out in January-February 2015 to gather information on how the Ebola crisis has affected the delivery and utilization of routine, non-Ebola-related RMNCH services. The selection of prefectures was deliberate, not random, and included all four geographic zones (Upper, Lower, Middle, and Forest) and a sample of prefectures that were “active,” “calm,” and “not affected” in relation to confirmed Ebola cases, as of November 2, 2014. Factors considered in the selection of the sites included travel time and feasibility of travel to the site for the data collection teams as well as input from advisors at USAID/Guinea and USAID/Washington.

Public facilities visited for data collection within selected prefectures included the central hospital (regional or district) and two health centers serving the nearby communities. Similarly, in Conakry, the medical centers from three city districts were selected, along with two health centers located in the areas around each of these. Overall, 12 prefectures and three city districts within Conakry were chosen for data collection. Private clinics serving the same catchment populations as the surveyed public facilities (within and outside of Conakry) were visited for health worker interviews whenever they could be identified.

Changes in the level of facility-based service delivery and utilization in public facilities were assessed through a facility record review. The record review focused on three service areas: family planning, maternal health, and child health, as well as overall outpatient visits, and spanned the months from October 1, 2013 to December 31, 2014 (for a total of 15 months). Indicators selected for assessment were based on the criteria of being an indicator for key RMNCH services, as identified by the Ministry of Health and Public Hygiene, and available at facilities and district health offices. The list of selected indicators is presented in Appendix 1. Private clinics were not included in the record review due to the differences in public and private record keeping.

Brief, structured interviews were conducted with: (1) health facility directors or managers (2) up to two providers of RMNCH services in each selected government facility; and (3) up to two providers in each private clinic. These interviews helped to document provider shortages/absenteeism, changes to service delivery, infection control practices, and provider safety concerns, among others. Interviews were conducted with providers who were available at the time of the interview, and who had served at the facility for at least one year prior to the survey. Interviews with traditional healers and Health District Officers were also conducted to collect information on the informal sector and overall service availability and the health labor force; these findings are reported elsewhere.^13^

Field work training, pretesting, and data collection were conducted in collaboration with StatView International, a private research firm based in Conakry. Six data collection teams, each including a physician, and three quality assurance teams were deployed. EPI Info 7 was used for data entry. Two data entry quality checks on each of the datasets were made before transferring the data into STATA v.13 for analysis.

Consent was obtained prior to initiating the interviews from all facility directors and and health care providers. No individual identifiers (e.g., name, address) were collected, and no sensitive questions related to personal health status were asked. Findings from individual clinics are not identified. Permission to undertake the survey was granted by the Guinea Ministry of Health and Public Hygiene (MOHPH). The activity was reviewed and received an exemption from the Institutional Review Board (IRB) at the University of North Carolina at Chapel Hill.

**Analysis**

Data on RMNCH service delivery and staffing were collected by month and aggregated into quarters. For each indicator, the percent change in the median number of services recorded between Quarter 1 (October 1 – December 31, 2013) and Quarter 5 (October 1 – December 31, 2014) was calculated and disaggregated by type of public facility, i.e., hospital/city district medical center (“hospital” hereafter) or health center.

The choice of the same annual quarters for comparison (i.e., the months of October through December one year apart) helped to account for seasonal variation in service provision. The Wilcoxon signed rank test was used to test for statistically significant differences between the indicator medians calculated for the two quarters (Q1 and Q5).

Trends over time using all 15 months of data were plotted for select indicators with statistically significant differences in Quarter 1 and Quarter 5 median numbers. Graphs were disaggregated by type of facility (hospital or health center) and Ebola status classification of the prefecture over the period of the survey. To classify the prefectures, the Ebola status of the prefectures over the nine-month period from March through December 2014 was reviewed and three categories were constructed: “Active” (prefectures that reported Ebola cases throughout the time period beginning in March 2014); “Inactive” (no diagnosed Ebola cases reported during the surveyed time period); and “Changing Status” (classification of prefecture varied—status classified as active, calm, and/or inactive during the surveyed period). These three categories were used to aid the interpretation of trends detected in indicators that showed a significant change over the period surveyed. Classification of selected prefectures and city districts was as follows: Active (Guèckèdou, and Conakry City Districts of Dixinn, Ratoma, and Matam), Changing Status (Boffa, Coyah, Dabola, Dalaba, Faranah, Fria, Kissidougou, N’Zèrèkorè, and Siguiri), and Inactive (Mamou and Mandiana).

Information from the facility director and provider interviews was analyzed and is reported as univariate distributions (frequencies). The bivariate distribution of responses between public and private providers was compared using a Chi-square test for equivalency, and only statistically significant results of the bivariate analysis are discussed. Responses to open-ended questions were reviewed and categorized by content and frequency. Selected portions of this qualitative information are reported to add insight into interpretation of the quantitative results.

## Results

**Facility Record Review**

A total of 45 public facilities were visited for record review, including 16 hospitals and 29 health centers. Most facilities were classified as being in Ebola “Changing Status” areas (n=26), while thirteen were in Ebola Active areas, and six were in Ebola Inactive areas. The median number of services provided for the selected indicators in the comparison time periods (October-December 2013 and October-December 2014) are shown in Table 1. Findings show that the median number of outpatient visits experienced a statistically significant decline between Quarter 1 and Quarter 5 in these hospitals (31%) and health centers (6%). The median number of family planning services recorded for the two time periods was low, and neither of the two indicators assessed, number of new acceptors to modern contraception and number of continuing users of modern contraception, showed statistically significant changes. Findings for maternal health indicators also suggest declines in routine services between the two time periods, though only one indicator, testing for HIV among pregnant women in surveyed hospitals, shows a significant decline of 51% between the two quarters.

On the other hand, many indicators for child health services showed significant declines between the two time periods. The median number of Pentavalent 1 and 3 vaccinations given at health centers declined 18% and 32%, respectively. The numbers recorded for Pentavalent 1 and 3 vaccinations at surveyed hospitals also showed declines, but these were not statistically significant. The median number of cases of malnutrition in children under age five recorded in health centers increased from two in Quarter 1 to nine in Quarter 5, a small but statistically significant change. The number of children under five seen for diarrhea and Acute Respiratory Infection (ARI) showed a large decrease over the one-year period in both hospitals (60% for diarrhea and 58% for ARI) and health centers (25% and 23%, respectively). The median number of children under five hospitalized for ARI declined throughout the surveyed period, but changes recorded between the baseline quarter and the final quarter were not statistically significant.

**Table 1:** Median Number of Services at 45 Public Facilities between Quarter 1 (Oct. – Dec. 2013) and Quarter 5 (Oct. – Dec. 2014): Facility Record Data, Guinea, 2015

Indicator	Median Number Quarter 1	Median Number Quarter 5	% change	Missing
Outpatient visits				
Hospitals	1355	930	-31**	2
Health centers	1223	1147	-6**	1
				
Family Planning				
New acceptors in hospitals	19	12	-37	1
New acceptors in health centers	25	34	+36	4
				
Continuing users in hospitals	60	50	-17	3
Continuing users in health centers	26	28	+6	4
				
Maternal Health				
Pregnant women tested for HIV in hospitals	112	55	-51*	7
Pregnant women tested for HIV in health centers	255	246	-4	15
				
Pregnancy complications in hospitals	10	8	-20	0
				
Deaths to pregnant women in hospitals	1	2	+100	1
Deaths to pregnant women in health centers	0	0	--	NA
				
Pregnant women seen for ANC 1 in health centers	337	295	-13	1
				
Pregnant women seen for ANC 3 in health centers	245	205	-16	2
				
Births in hospitals	303	281	-7	0
Births in health centers	100	69	-31	4
				
Child Health				
Penta 1 vaccinations given in hospitals	504	316	-37	13^a^
Penta 1 vaccinations given in health centers	259	212	-18**	3
				
Penta 3 vaccinations given in hospitals	353	320	-9	13^a^
Penta 3 vaccinations given in health centers	244	167	-32**	4
				
Cases of malnutrition in children <5 in hospitals	2	1	-50	5
Cases of malnutrition in children <5 in health centers	2	9	+500*	6
				
Cases of diarrhea in children <5 in hospitals	34	14	-60**	0
Cases of diarrhea in children <5 in health centers	16	12	-25**	0
				
Cases of ARI in children <5 in hospitals	98	41	-58**	2
Cases of ARI in children <5 in health centers	108	83	-23**	0
				
Hospitalized cases of ARI in children <5 in hospitals	18	16	-11	1

**p<0.01; *p<0.05

[a] This indicator was not originally intended to be collected at the hospital level, but the information was found at three sites and is included here for illustrative purposes.

The 15-month trends for select indicators with significant differences in median numbers, disagregated by whether the facility was located in an Ebola Active, Changing Status, or Inactive zone, are presented below. The full collection of trend graphs for indicators with significant differences in median numbers is reported elsewhere.^13^

**Outpatient visits**

When disaggregated by Ebola classification, it appears that surveyed hospitals located in Ebola Active areas had an increase in outpatient visits between Quarter 1 (October-December 2013) and Quarter 2 (January-March 2014), followed by steady declines in outpatient visits throughout the outbreak period of 2014 (Figure 1). Hospitals in the Ebola Inactive and Changing Status zones showed the largest declines in outpatient visits in the final Quarter of 2014, when the outbreak was at its peak.

**Trend by Ebola Active, Inactive, or Changing Status zone:**
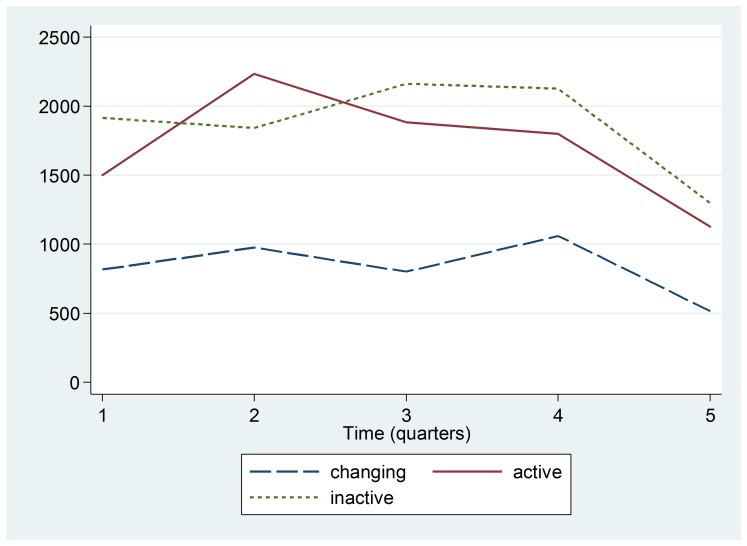
Median number of outpatient visits in hospitals

**Penta 3 vaccinations**

As detailed in Figure 2, declines in Pentavalent 3 vaccinations given at surveyed health centers are evident across all Ebola zones. The decline in the number of Pentavalent 3 vaccinations at these health centers began at the time of Ebola case detection, after Quarter 2 (January-March 2014), and was especially evident in Ebola Active zones.

**Trend by Ebola Active, Inactive, or Changing Status zone:**
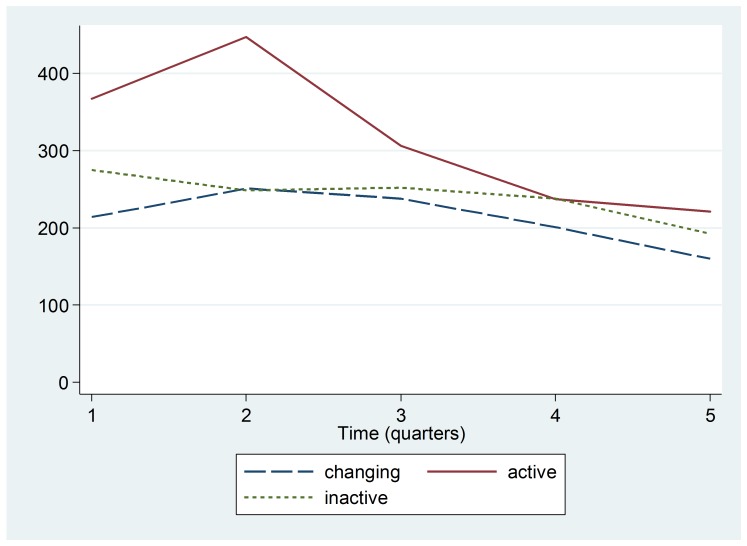
Median number of Pentavalent 3 vaccinations in health centers

**Diarrheal cases in children under five**

Figure 3 shows the trends for diarrheal cases in children under five in selected hospitals. The trends indicate that the median number of children receiving care for diarrhea declined in hospitals in all three Ebola status regions. However, the smallest amount of change was noted in the Ebola Changing Status areas.

**Trend by Ebola Active, Inactive, or Changing Status zone:**
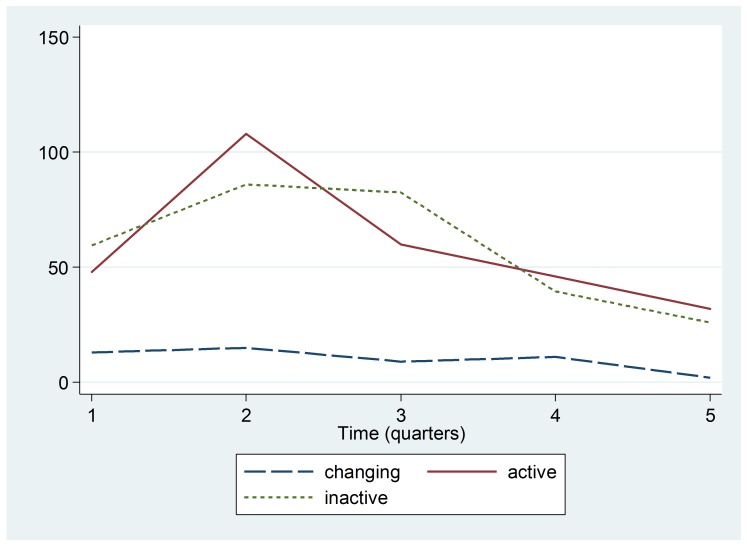
Median number of cases of diarrhea in children under five in hospitals

**ARI cases in children under five**

The trend lines shown in Figure 4 indicate that although there was an overall reduction in the median number of ARI cases in children under five in these selected health centers, there was actually an increase from Quarter 2 to Quarter 3 in ARI cases for centers located in Ebola Active regions.

**Trend by Ebola Active, Inactive, or Changing Status zone:**
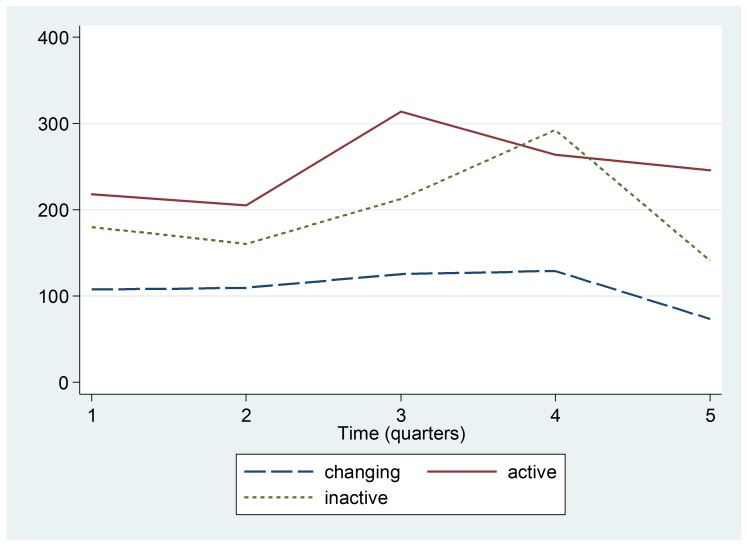
Median number of cases of ARI among children under five in health centers

**Stockouts**

Stockouts of contraceptives and key medications were fairly common throughout the study period (results not shown). At least one stockout of modern contraceptives (injectables, oral contraceptive pills, or condoms) was reported by 21% of facilities over the 15-month time period surveyed. However, only 5% of facilities reported that the stockout(s) occurred only during the time of the Ebola outbreak (April 2014 – December 2014).

Stockouts were more commonly reported for Oral Rehydration Salts (ORS) and Cotrimoxazole. Almost half of all surveyed facilities reported at least one stockout of ORS for the treatment of diarrhea (49%); for 18% of facilities the stockout(s) occurred only after the Ebola outbreak began, and not in the preceding months. Similarly, 40% of the surveyed facilities reported at least one stockout of Cotrimoxazole for treating ARI over the 15-month period, and for 20% the stockout occurred only after the Ebola outbreak began. Overall, these findings suggest that stockouts of these supplies and medications did not increase in the majority of facilities in the wake of the Ebola crisis.

**Interviews with Health Directors and Providers**

**Health Directors**

An additional 19 private clinics were included in the assessment of the effects of the Ebola crisis on facility operations, including changes in services and staffing issues, bringing the number of facilities included in this aspect of the assessment to 64. A total of 62 health directors/managers (“directors”) and 117 providers were interviewed.

The main quantitative findings from interviews with health directors are summarized in Table 2. The director reports indicated that provider absences during the Ebola crisis varied from 8% to 21% at their respective facilities, depending on staff type. Only a small proportion reported that their facility had to reduce hours of operation (13%), close (15%), or suspend services (13%). Just over one-quarter of directors (26%) reported that stockouts of medications or supplies increased during the Ebola crisis. Overall, the director responses suggest that the Ebola crisis has not seriously affected the availability of health care services across the surveyed facilities as a whole, but rather, that negative effects on access and service availability are likely to be regional and/or facility-specific.

Almost 20% of surveyed directors believed there had been an increase in complications due to delays in seeking care. While 76% reported the likelihood that community members had concerns about the safety of care at their facility, most also reported improved infection control practices at the facility: 68% of those interviewed reported having personally received training on Ebola infection control, and 85% reported that other providers at the clinic received such training. Eighty-five percent of directors reported having a screening/triage process for Ebola case identification, and 92% reported the implementation of additional infection control measures. Furthermore, most facility directors reported access to the supplies needed to prevent infection transmission, such as gloves (87%) and disinfectant (82%). Access to personal protective equipment was reported by 69% of the directors interviewed. Ninety percent of directors stated that their facility had been able to maintain routine data collection and reporting throughout the Ebola crisis.

**Table 2:** Service Availability and Health Labor Force Issues in Surveyed Facilities: Director Interview Data, Guinea, 2015 (N=62)

Facility Characteristics and Indicators	% Surveyed Directors
Facility management:	
Public	69
Private	31
Facility type:^	
Community Medical Center	5
Health Center	72
Hospital	23
Facility has had absence of:	
Doctors	15
Nurses	8
Auxiliary nurses/midwives	10
Other	21
Facility was unable to provide RMNCH services due to staff absences	10
Facility reduced hours due to Ebola crisis	13
Facility had closures due to Ebola crisis	15
Facility suspended services due to Ebola concerns	13
Type of service suspended at facilities with a service suspension:	
Syphilis test	6
Malaria rapid test	10
HIV test	6
TB test	3
Vaccinations/immunizations	3
Delivery services	6
Other	6
Facility experienced stockouts of medicines or supplies needed for routine RMNCH care beyond what was usual prior to Ebola crisis	26
Facility saw an increase in complications due to delays in seeking care	19
Director feels community members have concerns about safety of services at the facility	76
Director has personally received training on Ebola infection control	68
Director reports other providers at the facility have received training on Ebola infection control	85
Facility has implemented a screening/triage process for Ebola case identification	85
Facility has implemented other infection control measures as a result of Ebola	92
Facility has access to supplies needed for infection control:	
Gloves	87
PPE	69
Disinfectant	82
Hand Sanitizer	73
Other	26
Facility has been able to maintain routine data collection and reporting	90
Director is aware of negative reactions from family, friends, or community toward facility staff	45

^Missing (n=2)

In most cases, no statistically significant difference in response was observed by whether the director worked in a public versus a private facility. There were, however, a few responses that showed statistically significant differences (results not shown). These differences suggest that access to private clinics may have been more negatively affected by the Ebola crisis. Larger percentages of directors from private compared to public clinics reported reduced hours (32% compared to 5%) or suspended services (26% compared to 7%). Fewer providers at private facilities reported having been trained in Ebola infection control (68% compared to 93%). Interestingly, fewer directors in private facilities felt that the community had safety concerns about the services provided at their facility (58%) compared to public facility directors (84%). While data reporting systems differ widely between public and private facilities, public sector facilities appear to have been better able to maintain their systems during the Ebola crisis (74% of private and 98% of public).

**Service Providers**

A summary of quantitative responses from RMNCH provider interviews are presented in Table 3. The providers interviewed at the facilities included doctors, nurses, midwives, and other health care professionals. Three-quarters of providers were interviewed in public facilities, with almost half working in health centers. The majority reported learning of Ebola early in the outbreak period; only 15% reported learning of the outbreak after June 30, 2014. Seventy percent of providers reported that they had been trained in Ebola infection control and good practices for risk reduction. Nevertheless, the majority (80%) expressed concerns about the safety of service provision at their facility. Only a small percentage of surveyed providers reported that services had been suspended due to Ebola (services suspended included maternity care/delivery, small surgeries, nighttime consultations, and pediatric services). The vast majority of providers reported making changes to their service provision practices, including wearing gloves and other personal protective equipment; washing hands; taking patient temperatures; implementing safety protocols; and disinfecting the facilities. Just over one-quarter of the providers (28%) reported an increase in complications due to patient delays in obtaining services. Complications were especially noted in delivery/maternal care, severe anemia, and advanced malaria, especially among children.

**Table 3:** Service Availability and Practices in Surveyed Facilities: Provider Interview Data, Guinea, 2015 (N=117)

Provider Characteristics and Indicators	% Surveyed Providers
Provider type:	
Doctor	27
Nurse	30
Midwife	22
Other	21
Provider interviewed at:^	
Public facility	74
Private facility	26
Facility type:^	
Community Medical Center	5
Health Center	47
Private clinic	26
Hospital	22
Provider learned of Ebola in Guinea:	
Before April 1, 2014	65
April 1-June 30 2014	20
After June 30, 2014	15
Provider was trained in good practices for Ebola infection control and risk reduction	70
Provider reports concerns about the safety of health service provision at the facility	80
Provider reports that facility has had to suspend its provision of RMNCH services	7
Provider wears surgical gloves at each medical consultation, to draw blood or give vaccinations	97
Provider has changed own practices when delivering RMNCH services due to Ebola threat	96
Provider noticed an increase in complications among those who have delayed accessing RMNCH services	28
Provider noticed any other changes in service utilization during Ebola crisis	86
Provider perceived/noticed change in number of pregnant women living with HIV who received ART during pregnancy or delivery:	
Increased	15
Decreased	22
Remained the same	15
Don't know	47
Provider feels members of the community have or demonstrate concerns about the safety of service provision at the facility since Ebola crisis	63

^ Missing (n=32)

Most responses showed no statistically significant difference by public versus private facilities (results not shown). However, providers in private clinics were significantly more likely to a report suspension of services at their facility (23% compared to 1%), less likely to report noticing an increase in complications among patients who delayed seeking services (13% compared to 32%), and less likely than their public sector counterparts to report perceiving changes (increases or decreases) in the number of HIV-positive women accessing ART during pregnancy or delivery during the Ebola crisis (25% of private providers reported that numbers “stayed the same” as compared to 11% of public providers).

**Findings from Open-Ended Questions**

Health directors and service providers were asked a number of open-ended questions about negative reactions they experienced from family members, friends, or within their communities, in their role as health professionals during the Ebola crisis. Thirty-six percent of the RMNCH service providers interviewed, and 46% of the health directors interviewed, reported experiencing negative reactions, and provided the following examples of how those reactions were expressed or experienced:

“We are considered carriers of the Ebola.”

“People keep their distance from health workers; they mistrust us.”

“My daughter keeps away from me. My mother is suspicious.”

“The (local) vendor forbids me to enter his shop.”

“Our children are no longer accepted by their friends.”

“My neighbors don’t trust me.”

“I feel excluded.”

“Rocks have been thrown into the hospital’s courtyard.”

“Dr. Wamey was killed (by stoning, along with two others).”

“My family told me to leave Guèckèdou and not step foot in the hospital there.”

“My children have asked me to stop working.”

“I have been threatened to be burned by those in my community.”

“There was talk of destroying my clinic.”

“(Health) personnel are accused of spreading Ebola for money.”

Directors and service providers were also asked open-ended questions about what they perceived to be the most pressing problems they faced as health care professionals in the context of the Ebola crisis, and what was needed to address those problems. The following list extracts and summarizes the needed steps most commonly suggested, in order from highest to lowest frequency:

1. Maintenance of Ebola infection control measures

2. Stronger public health education messages

3. More secure supply of Personal Protective Gear

4. Response to fear, misgivings, and distrust shown toward health workers

5. More/improved Ebola trainings for health workers

6. Hardship pay for health workers in Ebola-stricken areas

## Conclusions

This assessment of RMNCH service delivery and utilization was based on data abstraction and brief structured interviews at a selection of health care facilities in 12 prefectures and three city districts in Guinea from January to February 2015. Quality control measures implemented during data collection helped to ensure that the information collected was as complete and accurate as was feasible.

Though not necessarily representative of the country as a whole, the assessment in Guinea revealed a number of important findings. First, there was an overall decline in service utilization, as seen in the median numbers of outpatient visits in facilities participating in the assessment. The decline was especially notable in hospitals (which saw a 31% reduction in the median number of outpatient clients from October – December 2013 to October – December 2014). Though not always statistically significant, declines for specific RMNCH services were also greater in hospitals than in health facilities. This finding suggests that hospital services suffered more from Ebola-related stigma than did services at health centers.

Second, child health services, such as vaccinations, were more affected by the Ebola epidemic than other assessed health areas. Significant declines in the number of cases of diarrhea and ARI were also recorded. These findings suggest that parents may have been reluctant to bring children to health facilities out of fear of contracting Ebola. However, it is also possible that declines in diarrhea cases over time could be due to improved access to clean water and systematic hand washing by care givers. It was noted, however, that the number of cases of ARI in health centers in Ebola active zones increased during the Ebola outbreak. The significant rise in cases of child malnutrition, though not common, is a concern; and median numbers were especially high for facilities in Ebola Active zones. Related to this, provider interviews also suggest childhood anemia may be on the rise.

Third, it appears that the Ebola epidemic has not had a widespread negative effect on the availability of health care services. Results suggest that the negative effects on service availability (such as reduced hours, closures, and service suspensions) are likely to be regional and/or facility-specific. Additionally, declines in service availability appeared to be more common in private facilities than in public facilities.

Fourth, providers reported a number of improved infection control behaviors as a result of the Ebola outbreak, including more frequent hand-washing and the use of disinfectants. These behaviors must be encouraged to ensure they continue throughout and beyond the current Ebola crisis. Nevertheless, 30% of interviewed staff had not received any training on Ebola infection control. This gap likely hindered government efforts to control the epidemic, and potentially further exposed the health staff to Ebola disease.

Finally, there are a number of negative social consequences to being a health care worker during the Ebola epidemic. These consequences represent a serious concern for safety, epidemic control, and the maintenance of family and social networks.

A main limitation to this assessment was that the prefectures and facilities were chosen on the basis of particular criteria, rather than by random selection, and findings were therefore susceptible to sampling bias. For example, the sampling favored facilities that were more easily accessible by road and, in the forest region, that were located in communities in which no violent acts toward Ebola medical workers had occurred (as a safety precaution to the data collection teams). Thus, the impact of Ebola on RMNCH services in the facilities not included in the survey may be different than what is reported here. Recent findings from a study on the effect of Ebola on malaria treatment in Guinea, which used stratified random sampling, reported similar levels of declines in all-cause outpatient visits (11%), cases of fever (15%), and patients treated with oral (24%) and injectable (30%) antimalarial drugs.^14^ Such findings strengthen the external validity of results reported here.

Another limitation is that the assessment relied on data from facility registers and self-reports from health workers. No attempt was made to verify the accuracy of either sources of data. However, it is assumed the level of inaccuracy is constant and could allow comparison of indicators over time.

Despite these limitations, this is the first assessment of the effect of Ebola on RMNCH services during the current outbreak. A large number of RMNCH service indicators were assessed from different sources in order to provide a detailed snap-shot of the situation and provide needed information for programming and health system response. The information is therefore an important and timely contribution to on-going efforts to understand and respond to the Ebola crisis in Guinea.

## Competing Interest

The authors have declared that no competing interests exist.

## Appendix 1: Indicators

**Family Planning**

Number of new acceptors to modern contraception

Number of continuing users of modern contraception

Number of facilities that experienced a stockout of a modern contraceptive method (disaggregated by type: injectables, pills, condoms)

**Maternal Health**

Health Center

Number of pregnant women tested for HIV

Number of pregnant women seen for first antenatal visit (ANC 1)

Number of pregnant women seen for scheduled visit during last trimester (ANC 3)

Number of deaths to pregnant women occurring in the facility

Number of births occurring in the facility

Hospital

Number of pregnant women tested for HIV

Number of cases of pregnancy related complications (eclampsia, uterine rupture, or complication of abortion)

Number of deaths to pregnant women occurring in the hospital

Number of births occurring in the hospital

**Child Health**

Health Center

Number of Pentavalent 1 vaccinations given

Number of Pentavalent 3 vaccinations given

Number of cases of malnutrition in children under 5

Number of watery/bloody diarrhea cases in children under 5

Number of facilities that experienced stockouts of oral rehydration salts

Number of facilities that experienced stockouts of antibiotics for the treatment of ARI (cotrimoxazole)

Number of cases presenting acute respiratory infection for children under 5

Hospital

Number of hospitalized cases of ARI in children under 5

**Facility**

Number of outpatient visits/facility (children and adults)
